# Robust Intensity Modulated Proton Therapy (IMPT) Increases Estimated Clinical Benefit in Head and Neck Cancer Patients

**DOI:** 10.1371/journal.pone.0152477

**Published:** 2016-03-31

**Authors:** Lisanne V. van Dijk, Roel J. H. M. Steenbakkers, Bennie ten Haken, Hans Paul van der Laan, Aart A. van ‘t Veld, Johannes A. Langendijk, Erik W. Korevaar

**Affiliations:** 1 Department of Radiation Oncology, University of Groningen, University Medical Center Groningen, Groningen, The Netherlands; 2 Institute for Biomedical Technology and Technical Medicine (MIRA), University of Twente, Enschede, The Netherlands; Taipei Medical University, TAIWAN

## Abstract

**Purpose:**

To compare the clinical benefit of robust optimized Intensity Modulated Proton Therapy (minimax IMPT) with current photon Intensity Modulated Radiation Therapy (IMRT) and PTV-based IMPT for head and neck cancer (HNC) patients. The clinical benefit is quantified in terms of both Normal Tissue Complication Probability (NTCP) and target coverage in the case of setup and range errors.

**Methods and Materials:**

For 10 HNC patients, PTV-based IMRT (7 fields), minimax and PTV-based IMPT (2, 3, 4, 5 and 7 fields) plans were tested on robustness. Robust optimized plans differed from PTV-based plans in that they target the CTV and penalize possible error scenarios, instead of using the static isotropic CTV-PTV margin. Perturbed dose distributions of all plans were acquired by simulating in total 8060 setup (±3.5 mm) and range error (±3%) combinations. NTCP models for xerostomia and dysphagia were used to predict the clinical benefit of IMPT versus IMRT.

**Results:**

The robustness criterion was met in the IMRT and minimax IMPT plans in all error scenarios, but this was only the case in 1 of 40 PTV-based IMPT plans. Seven (out of 10) patients had relatively large NTCP reductions in minimax IMPT plans compared to IMRT. For these patients, xerostomia and dysphagia NTCP values were reduced by 17.0% (95% CI; 13.0–21.1) and 8.1% (95% CI; 4.9–11.2) on average with minimax IMPT. Increasing the number of fields did not contribute to plan robustness, but improved organ sparing.

**Conclusions:**

The estimated clinical benefit in terms of NTCP of robust optimized (minimax) IMPT is greater than that of IMRT and PTV-based IMPT in HNC patients. Furthermore, the target coverage of minimax IMPT plans in the presence of errors was comparable to IMRT plans.

## Introduction

In Head and Neck Cancer (HNC) patients, radiation-induced side effects, in particular xerostomia and dysphagia, have a major impact on quality of life [1–3]. In the last decades, Intensity Modulated Proton Therapy (IMPT) has developed as a treatment modality to reduce these side effects in HNC patients [4–8]. Unfortunately, IMPT can be more sensitivity to uncertainties in patient setup, CT values and patient anatomy than intensity modulated radiotherapy (IMRT) as radiologic path length changes result in displacements of the steep Bragg peak fall-off. In particular, range errors, which arise from inaccuracies in the planning CT and the CT Hounsfield units-to-stopping power calibration curve, are an issue in proton therapy [9–11]. Currently, in both IMRT and IMPT, these uncertainties are commonly taken into account by expanding the clinical target volume (CTV) to the planning target volume (PTV) to ensure adequate dose coverage of the CTV [12,13]. However, the physical properties of protons can conflict with this traditional CTV-PTV concept, as errors can result in centralized target volume under-dosage, ultimately risking tumor recurrence [11,14–19]. Therefore, proton therapy requires a different approach to achieve robustness, which refers to the correspondence of planned and actual dose distributions in presence of errors and unanticipated changes. Park et al. [20] showed that a field-specific PTV was beneficial for single field uniform dose (SFUD), where each field delivers uniform dose to the target volume. However, IMPT requires a more complex integration to achieve robustness.

Including robustness in the optimization has been proposed as strategy by several authors to reduce the impact of potential errors [14,15,21]. Fredriksson et al. [22] has developed a worst case scenario optimization (i.e. minimax optimization), that penalizes the perturbed dose distributions that are the most unfavorable by minimization of the worst objective function value that corresponds to one scenario. Dealing with worst case physically realizable dose distributions, makes this approach less conservative [10] and computationally more demanding than other robust optimization implementations [14].

Multiple studies have shown the potential benefits of IMPT for HNC patients by comparing proton therapy with photon modalities [4,5]. However, these comparisons were potentially not fair, because the used IMPT plans were PTV-based and may, therefore, lack robustness. In the head and neck regions the presence of multiple nearby Organs At Risk (OARs) that are preferably spared as much as possible makes HNC plans complex. It is therefore especially important in these patients to incorporate robustness in the optimization process [23]. However, just as important is the evaluation of the robustness of these plans by examining the target coverage in presence of setup and range errors. No anatomic deformations were included in our study.

Our study is the first to evaluate IMRT, PTV-based IMPT and robust optimized IMPT plans not only in terms of dose to OARs and estimated clinical benefit in terms of normal tissue complication probabilities (NTCP), but also on robustness in terms of target coverage and NTCP. Furthermore, all treatment plans were generated using the same treatment planning system. This allows comparison of the different modalities without introducing biases related to the use of different planning systems [19]. Since the number of fields in robust planning might influence the dose to OARs [18] or the robustness of the IMPT plans [11,14,17], this aspect was also included in our study.

## Methods and Materials

### Patients and inclusion criteria

For this study, 10 HNC patients with varying tumor sites, extensions and regional lymph node status were included (Table 1). All patients were treated bilaterally with parotid and swallowing sparing IMRT [24].

**Table 1:** Patient characteristics.

	Tumor location	Staging	Nodal Levels left and right		CTV_70_ volume (cm^3^)
1	Glottis larynx	T3N0	II-VI	II-VI	15
2	Oropharynx	T3N2b	Ib-V*	Ib-IV*	195
3	Oropharynx	T4N2c	Ib-V*	Ib-V*	157
4	Nasopharynx	T4N1	Ib-V*	II-III	154
5	Nasopharynx	T2N3	Ib-V*	Ib-V*	326
6	Supraglottis Larynx	T3 N2c	Ib-V	Ib-V	100
7	Oropharynx	T3 N0	II-IV	Ib-IV	80
8	Oropharynx	T2N2b	II-IV	I-V*	96
9	Oropharynx	T1N0	II-IV	II-IV	22
10	Oropharynx	T2N0	II-IV	II-IV	43

*retrostyloid, retropharyngeal

### Ethics Statement

All patients provided written informed consent before starting therapy that their data could be used within the department’s research program. Some of the authors were directly involved in treating patients and had access to identifying patient information. All data was anonymized by one of the authors (LD) and collected as part of a prospective data registration program within the framework of routine clinical practice. The Dutch Medical Research Involving Human Subjects Act is not applicable to data collection as part of routine clinical practice and therefore, the hospital ethics committee granted us a waiver from needing ethical approval for the conduct of studies based on these data. The treatment planning reported in this retrospective study was not used for actual patient treatment. All patients received the standard clinical practice of treatment with IMRT.

### IMRT and IMPT plan specifics

The clinical 7-field IMRT plans were optimized in the RaySearch treatment planning system (RayStation version 3.99) for 6 MV photon beams of an Elekta linear accelerator equipped with an MLC with 10 mm leaf width. All IMRT treatments applied a simultaneous integrated boost (SIB).

IMPT plans were constructed using 4 different field configurations (Table 2) in the RaySearch treatment planning system. The initial beam energy ranged between 70 and 230 MeV for an IBA dedicated gantry with a spot size in air of 3 mm at highest energy (one sigma). Range shifters of 40 mm water equivalent thickness were available.

**Table 2:** Field configurations used for IMPT and IMRT plans.

Type	Gantry angles (degree)						
**2F-IMPT**	50°	310°					
**3F-IMPT**	50°	310°		180°			
**5F-IMPT**	50°	310°	115°	180°	245°		
**7F-IMPT**	0°	50°	100°	150°	210°	260°	310°
**7F-IMRT**	0°	50°	100°	150°	210°	260°	310°

The IMPT configurations were applied for both PTV-based and minimax optimization. Gantry angles according to IEC 61217.

#### PTV-based IMRT plans

In the clinical plans of the patients included in this study the CTV to PTV margin was 5mm. With on-line cone beam CT setup correction tighter margins are feasible so for this study the criterion was used that plans needed to be robust against setup errors of 3.5 mm and PTVs were reduced accordingly [25]. Prescribed doses of 70 Gy and 54.25 Gy were conformed to the targets PTV_70_ and PTV_54.25_, respectively. The minimum target dose requirement was 95% of the prescribed dose in ≥ 98% of the PTVs. Furthermore, besides critical structures such as brain and spinal cord tissue, parotid gland and swallowing related organs (S1 Fig) were spared as much as possible [24].

#### PTV-based IMPT plans

PTV-based IMPT plans were constructed with identical OAR objectives as IMRT plans, as described earlier [4]. However, as dose can be better conformed to PTVs in proton therapy, OAR objectives could be set to lower doses or higher weights (S1 Table), whilst keeping adequate target coverage. Moreover, a PTV margin of 5 mm was used to instead of 3.5 mm, because this could potentially account for both the range and setup errors in proton plans. PTV-based optimization settings were kept identical for all field configurations per patient, to prevent bias in the robustness analysis as function of field quantity.

#### Minimax IMPT plans

Minimax optimization aims to create robust IMPT plans by incorporating robustness into the optimization process [22]. Hereby, CTVs are targeted instead of PTVs. Minimax IMPT plans were constructed with identical OAR objectives as for PTV-based IMPT plans in all 5 field configurations, but again they could be set to lower doses or weights. Since these plans target the CTV instead of PTV, all related objectives were replaced. The minimum CTV dose requirements were increased by approximately 1.5–2 Gy. This was necessary to achieve similar coverage of the CTV in minimax IMPT plans compared to PTV-based IMRT and IMPT plans in the nominal situation. Potential errors were taken into account by simulating different error scenarios. Range errors were simulated by proportionally changing CT values by ± 3% [9,26]. Setup errors were incorporated by rigidly shifting the isocenter of the beams in six isotropic orthogonal directions. To mimic the PTV margin of 3.5 mm, a displacement of 3.5 mm was used as a magnitude of systematic setup errors for the robustness settings. The simulated error scenarios included combinations of range and setup scenarios and result in 21 error scenarios. Robustness is incorporated in the plans by optimizing the maximum objective function value of error scenarios including target and OARs objectives in these error scenarios. To avoid convergence problems due to discontinuity of the gradient of the objective function, the values of the other (not worst case) error scenarios are included with small weights [27].

#### Evaluation of robustness

To evaluate plans for robustness, multiple possible range and setup error combinations were simulated to investigate large variability of error scenarios in patients. No anatomic deformation were included in our analysis. Range errors are simulated by proportionally changing CT intensity values (+/-3%).The effect of setup errors was assessed by shifting the isocenter isotropically in 26 directions on the radius of a sphere with a radius of 3.5 mm. It should be noted that in the evaluation of robustness more shifts were considered than in the minimax optimization, where only 6 non-diagonal shifts were considered. The robustness criterion is only met if none of all error scenarios causes under-dosage to the CTV.

For all 10 patients, the combined systematic setup and range errors ultimately resulted in 6240 and 260 perturbed dose distributions of IMPT and IMRT plans, respectively. These were all compared to their corresponding planned, i.e. nominal dose distribution.

### Evaluation measures

To meet the robustness criterion, CTVs were tested to receive acceptable target coverage in all perturbed dose distributions. CTV coverage was acceptable if the dose that 98% of the volume (D_98_) received was at least 95% of the prescribed dose (CTV_70_: D_98_>66.5 Gy; CTV_54.25_: D_98_>51.5 Gy). Furthermore, target homogeneity (D_5_– D_95_) and hotspots (D_2_ and D_5_) were considered.

To estimate the clinical benefit of using minimax IMPT in comparison to conventional IMRT, Normal Tissue Complication Probability (NTCP) models for xerostomia and dysphagia were used. The risk of xerostomia was estimated using the model described by Houweling et al. [28]. In that study, xerostomia was defined as a minimal reduction in salivary flow (ml/min) to 25% of baseline level. For dysphagia, a multivariate regression NTCP model was used to estimate grade 2–4 RTOG swallowing dysfunction 6 months after RT [29]. Both mean doses of supraglottic larynx and superior pharyngeal constrictor muscle were individual input variables for this model. Additionally, NTCP values of IMRT and minimax IMPT were compared in the worst case error scenario.

## Results

### Nominal CTV coverage

In the nominal (non-error) scenarios, all CTVs were adequately covered in all PTV-based plans and minimax plans. Average nominal CTV D_98_ of all patients and field configurations were comparable for all investigated modalities (Table 3). Nominal target dose homogeneity was comparable in IMRT and minimax IMPT plans and somewhat more homogeneous in PTV-based IMPT plans (Table 3). The lower target dose homogeneity in nominal scenario minimax IMPT plans was primarily due to a D_5_ increase for CTV_70_. However, no objectionable hotspots were created in CTV_70_ in plans with more than 2 fields (D_5_< 73.6 Gy and D_2_<74.2 Gy in all patients). In error scenarios, PTV-based plans became more inhomogeneous compared to IMRT and minimax IMPT. In minimax plans with 2 fields the maximum dose was generally higher (D_5_< 73.9 Gy and D_2_<75.4 Gy). Maximum dose was observed in CTV_70_.

**Table 3:** Average CTV coverages and homogeneities per modality of all included plans.

Target	Modality	Nominal D_98_	Perturbed ${\text{D}}_{98}^{\text{min}}$	Nominal D_5_ − D_95_	${\text{Perturbed}\;\text{D}}_{5}^{\text{max}}-{\text{D}}_{95}^{\text{min}}$
	IMRT	68.9± 0.5	67.7± 0.8	3.3± 0.9	4.8± 0.7
**CTV**_**70**_	PTV-based IMPT	68.6± 0.4	64.4± 2.4	2.4± 0.5	7.4± 2.3
	Minimax IMPT	68.5± 0.2	67.5± 0.5	4.2± 0.4	5.8± 0.6
	IMRT	54.0± 0.8	53.2± 0.8	17.2± 1.0*	18.5± 0.9*
**CTV**_**54.25**_	PTV-based IMPT	53.5± 0.8	50.5± 1.4	16.9± 1.0*	20.8± 1.7*
	Minimax IMPT	53.7± 0.6	52.7± 0.3	17.7± 0.9*	19.1± 0.8*

Values are in Gy and averaged over all patients and (2,3,5,7) field configurations for the IMPT plans with their standard deviation (±).

*These values were obtained for CTV_54.25_ that contains CTV_70_. These values are not corrected for the simultaneous integrated boost.

Abbreviation: D_98_, dose that 98% of the CTV minimally receives; nominal, planned; perturbed, modeled with range and setup errors

### Plan robustness for target coverage

**Fig 1:**
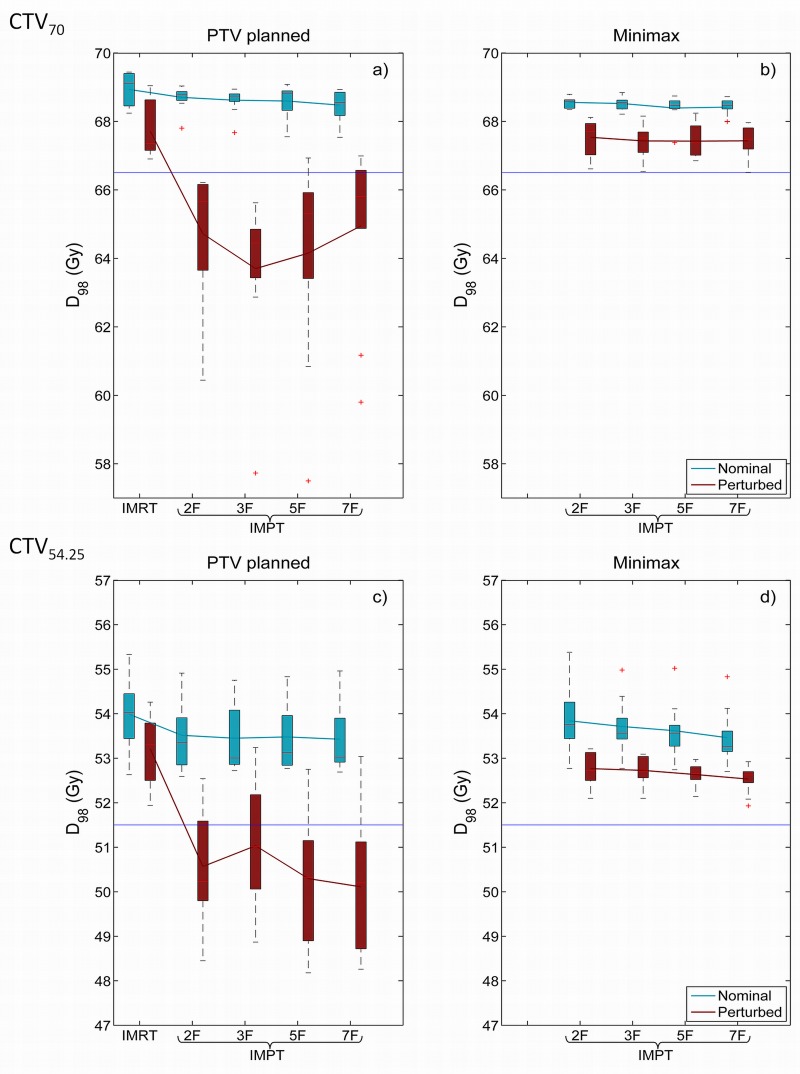
Robustness performance of PTV and minimax optimization. Nominal D_98_ (blue) and worst-case scenario ${\text{D}}_{98}^{\text{min}}$ (red) of PTV-based (a,c) and minimax IMPT plans (b,d) for CTV_70_ (a-b) and CTV_54.25_ (c-d). Solid lines connect the averages and boxplots represent the distribution of these parameters of all patients per plan type. The horizontal blue lines represent the CTV dose criterion that marks 95% of the prescribed dose (66.5 Gy and 51.5 Gy).

**Fig 2:**
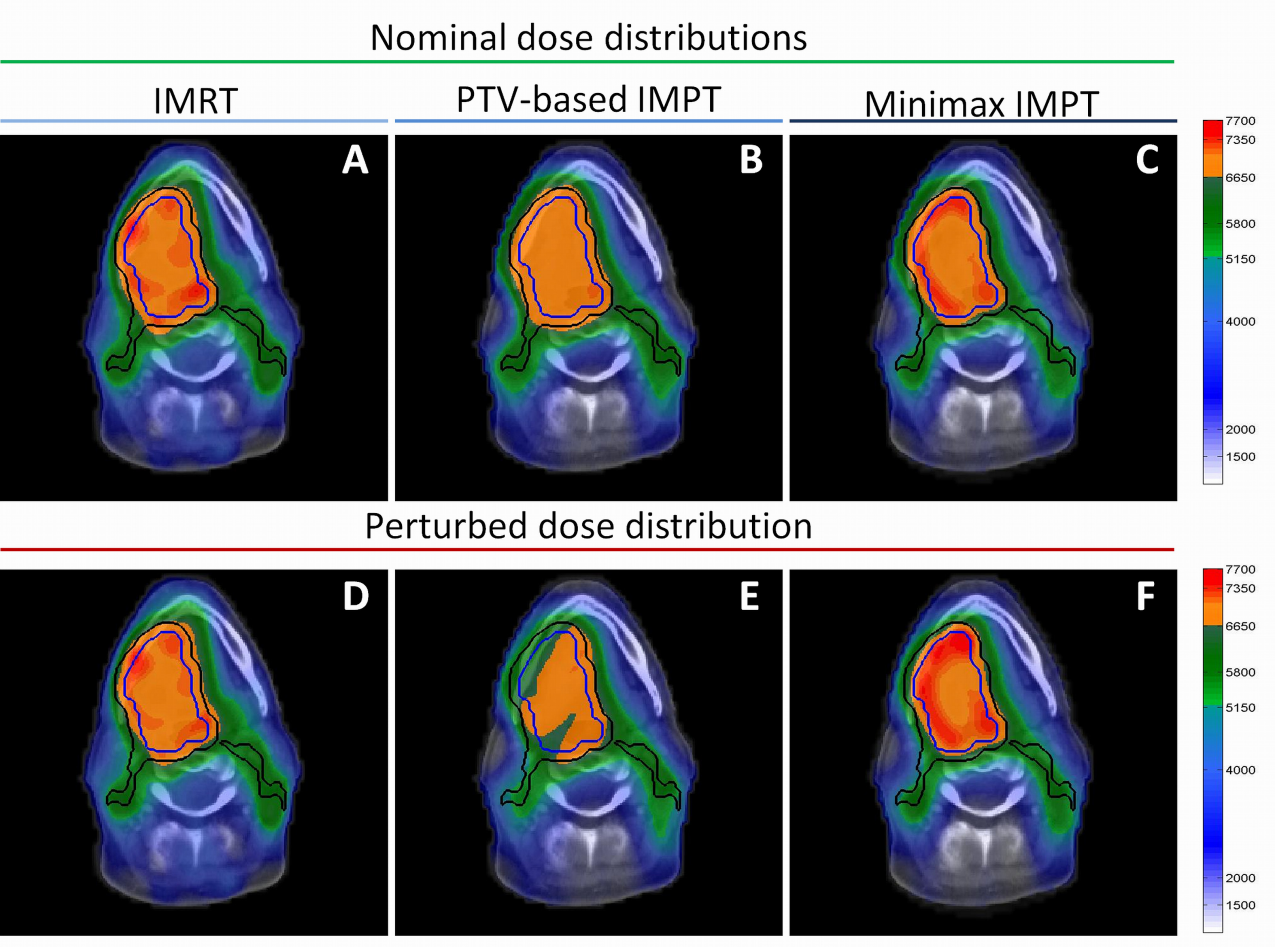
Example robustness performance in nominal and error scenario. Dose distributions of IMRT (a,d), PTV-based IMPT (b,e) and minimax optimized IMPT plans (c,f) in nominal (a-c) and an error scenario (d-f) with a setup error of x = 0.25;y = 0;z = 0.25cm and a range error of 3%. Both CTV_70_ (blue lines) and CTV_54.25_ (black lines) are shown in all dose distributions. IMPT plans were configured with 3 fields.

As expected, all IMRT plans met the robustness criterion, as minimal perturbed D_98_’s (${\text{D}}_{98}^{\text{min}}$) of all patients remained above the threshold of the robustness criterion in the presence of systematic errors (Fig 1A and 1C). The same holds true for minimax IMPT plans for all field configurations (Fig 1B and 1D). Average perturbed ${\text{D}}_{98}^{\text{min}}$’s of minimax and IMRT were comparable (Table 3). In contrast, a large fraction of the perturbed ${\text{D}}_{98}^{\text{min}}$ for PTV-based IMPT plans failed to meet the criterion. Acceptable target coverage of CTV_70_ and CTV_54.25_ was only ensured in 4 and 9 out of 40 plans, respectively. Moreover, for both CTVs, this was only in 1 out of 40 plans. This corresponds to ${\text{D}}_{98}^{\text{min}}$ averages that were below the robustness criterion (Table 3). Insufficient robust PTV-based plans were seen in patients with HNC in all included anatomic subsites as listed in Table 1. Moreover, visual evaluation of the perturbed dose distributions showed centralized cold spots in the CTV_70_ (Fig 2). Additionally, target dose homogeneity was more stable in minimax than in PTV-based IMPT plans, as the perturbed homogeneity values (${\text{D}}_{5}^{\text{max}}-{\text{D}}_{95}^{\text{min}}$) were lower in minimax plans.

Furthermore, whereas increasing field numbers did not have much effect on plan robustness in minimax plans, in PTV-based IMPT plans it appeared to have a somewhat a negative effect on CTV_54.25_ coverage (Fig 1).

### Benefit of minimax IMPT compared to IMRT

**Fig 3:**
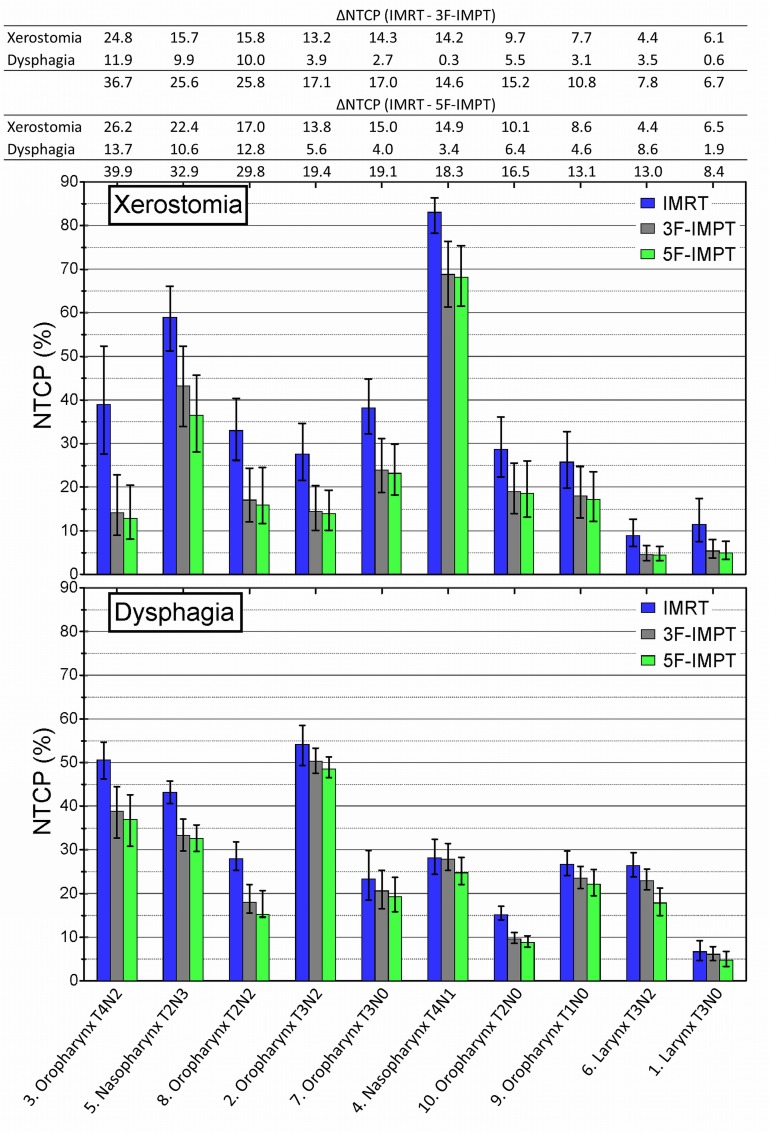
Estimated normal tissue complication probabilities (NTCP). NTCP values of xerostomia (upper) and dysphagia (lower) are shown per patient for IMRT (blue), 3-field minimax IMPT (gray) and 5-field minimax IMPT (green). ∆NTCP values comparing IMRT and 3 (upper table) or 5 fields (lower table) of xerostomia and dysphagia are shown for every patients. A summation of these ∆NTCP are also given, and patients are sorted accordingly. Error bars indicate NTCP values in worst and best case error scenarios.

Comparing minimax IMPT to IMRT, there is a potential clinical benefit in terms of estimated xerostomia and dysphagia NTCP values in all patients. However the magnitude of this benefit varied among the individual HNC patients included in this study (Fig 3). Seven patients had relatively large estimated improvements (∆NTCP) with 5 field configurations. These patients had a reduction of 10% for at least one of the evaluated complication probabilities and more than 15% reduction of xerostomia and dysphagia NTCP values together. The summation of NTCP of the individual patients are depicted in Fig 3. On average, NTCP reductions were 17.0% (95% CI; 13.0–21.1) for xerostomia and 8.1% (95% CI; 4.9–11.2) for dysphagia in these 7 patients.

The remaining 3 patients had average estimated reductions of 6.5% (95% CI; 4.2–8.8) for xerostomia and 5.0% (95% CI; 1.2–8.8) for dysphagia. The NTCP values for xerostomia were already low with IMRT in the two patients with laryngeal cancer and the patient with a T1N0 oropharyngeal cancer. Therefore, the absolute reduction of these values remained relatively small.

**Fig 4:**
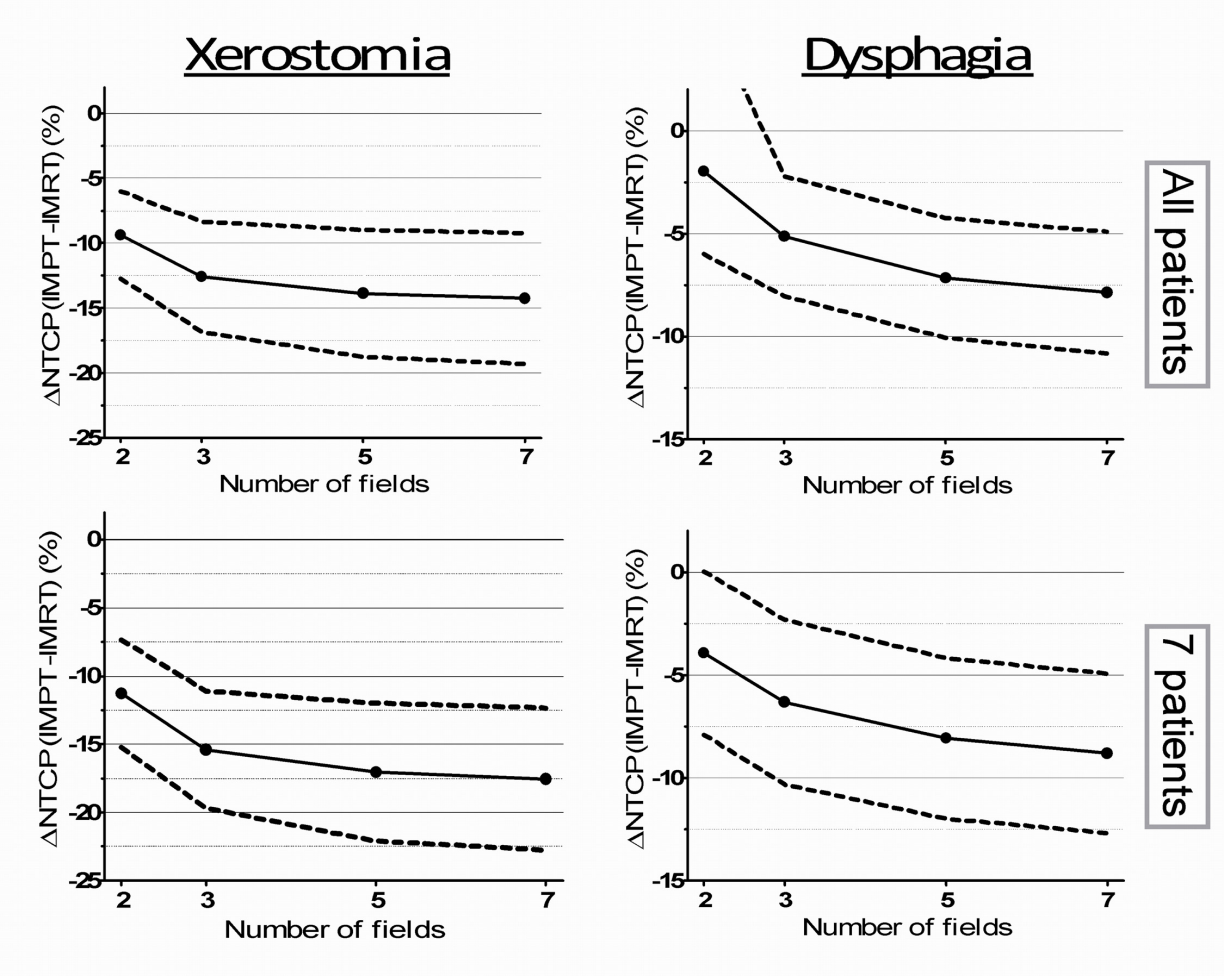
NTCP benefit of IMPT with different field configurations compared to IMRT. ∆NTCP and 95% confidence interval are given as a function of number of minimax-optimized IMPT fields for xerostomia (left), dysphagia (right), for all patients (upper) and the 7 patients with combined NTCP reduction larger than 15% (lower).

The use of a 5-field instead of a 3-field configuration resulted in lower NTCP values in all patients (Fig 3). It can be seen from Fig 4 that increasing the number of field improves the sparing of healthy tissue, but that the benefit does not increase linearly. In other words, the estimated benefit in terms of ∆NTCP still improves going from 5 to 7 fields, but not in the same magnitude going from 2 to 3 or 3 to 5 fields.

Estimated NTCP reductions in the worst case scenarios of minimax IMPT compared to IMRT for xerostomia and dysphagia were 17.1% (95% CI; 11.4–22.7) and 8.2% (95% CI; 6.1–10.4) in the most favorable 7 patients, respectively. These reductions were very comparable to those in the nominal scenario. The patient individual NTCP differences of IMRT, 3 field and 5 field minimax IMPT plans are also comparable in both worst and best case error scenarios, which is depicted by error bars in Fig 3.

## Discussion

Our results demonstrate that selected HNC patients can benefit greatly from robust optimized IMPT compared to IMRT in terms of estimated xerostomia and dysphagia NTCP values. Moreover, our study showed that minimax optimization for IMPT is comparably robust for systematic setup and range errors as the commonly applied PTV concept for IMRT. The minimax IMPT plans had adequate target coverage (D_98_> 95% of prescribed dose) of the CTVs in all 3120 simulated error scenarios of these plans. Additionally, the NTCP benefit of IMPT to IMRT remained similar in worst-case error scenarios, indicating that the investigated OARs are not more sensitive to errors with IMPT.

In contrast, the PTV concept for IMPT showed poor robustness performance in HNC patients, as acceptable target coverage in presence of errors was only obtained in 1 of 40 PTV-based IMPT plans. Since enlarging the PTV margin to 5 mm could not prevent coldspots in the border and center of CTVs, a margin concept seems inadequate for IMPT. These PTV robustness results complement previous studies, indicating unfavorable use of the PTV concept for IMPT [14,15,17]. However, the drastically poor robust performance of it that can be seen from our result has to our knowledge never been shown to such an extent in HNC. This performance was not improved by the use of different field configurations.

Although IMRT and minimax IMPT are comparable in terms of robustness, they are generally not comparable in terms of estimated NTCP values. Most of the patients included in this study could benefit greatly from IMPT, because of the relatively large NTCP changes. However, this was not the case for all HNC patients. This argues for a careful selection procedure of HNC patients that benefit from IMPT. Therefore, we consider individualized analysis to be essential for the selection of HNC patients for proton therapy [30]. Although the threshold of >15% combined xerostomia and dysphagia NTCP reduction to select the best HNC patients was arbitrarily chosen, we believe that through such a method patients can be selected that can have the most benefit of proton therapy [31]. It is noteworthy that use of different NTCP models may result in different estimated benefits. Current NTCP models are developed on patient populations that are radiated with IMRT and they may be different when dose was delivered with IMPT.

The estimated clinical benefit of IMPT that was shown by the studies that compared IMRT and PTV-based IMPT plans [5,32] could have potentially been greater by using robust optimization instead of PTV-based IMPT plans. This was shown by the study of Stuschke et al. [23]; robust IMPT plan optimization may lead to a decrease in dose to body volume and OARs (ipsilateral temporal lobe, cerebellum and brainstem) in HNC patients. Our findings complemented this, but were kept outside the descriptive scope of this study.

Increasing the number of field directions did not significantly improve robustness for PTV-based IMPT, which is in line with findings of Kraan et al. [17]. In fact, increasing field numbers resulted in fewer patients with acceptable perturbed CTV_54.25_ coverage. This is probably due to the increased conformity of dose to the target volume. Minimax IMPT plans did not show specific field dependency of the plan robustness considering both target volumes. Instead, a relationship between increasing field number and a decrease in mean dose to OARs was observed, which also translated to reduced NTCP estimates. Especially increasing two to three field numbers lowered the dose to OARs substantially, which was also seen by Hopfgartner et al. [18].

We aimed at a realistic choice of setup and range errors in the robust optimization and evaluation in this study but it can still be argued that they are too small or too large. Techniques like cone beam CT and dual energy CT are not commonly available in proton clinics but can be expected to be standard practice in the near future [33]. In this work, the choice of setup and range errors in robust planning is therefore somewhat arbitrary and is subject to change depending on development of available imaging and verification techniques at proton treatment clinics. Secondly, the PTV margin for the proton plans was enlarged with 1.5 mm, in an attempt to account for the range error, resulting from CT inaccuracies. Although this is also arbitrarily chosen, we believe that enlargement of the PTV margin would not improve the results sufficiently, as in IMPT plans errors will still occur in the center of the CTV. Next to the CTV central located errors, also the errors are often located at the border of the target volume. Therefore, we believe that reducing the PTV will influence the robustness even more.

Even though both IMRT and minimax IMPT modalities are robust, not including robust optimization for IMRT plans and by using a 10 mm leaf width instead of 5 mm might have overestimated the benefit in terms of NTCP values of IMPT somewhat [34]. Nevertheless, we expect that robust optimization for IMRT will not eliminate the estimated significance of IMPT for well selected HNC patients, because also the minimax IMPT plans could be improved. In contrast to IMRT, the optimization time of minimax IMPT is long (~ 1 hour), making it impracticable to iterate the optimization procedure to find the optimal plan. Furthermore, not included in the robustness optimization and evaluating are random setup errors, which blur dose distributions by changing inter-fractionally. In the PTV concept the random errors are taken into account by enlarging the margin slightly [35], we expect that the same can apply for using a slightly enlarged setup error as input for the robust optimization. Further research is necessary to investigate if indeed the impact of random errors for IMPT is similar to that for IMRT in HNC patients. The same applies to rotational displacements and anatomic deformations. We do believe that some of these non-rigid displacement errors could partly be accounted for by robust optimization, since fixation techniques (thermoplastic mask and headrest) ensure a minimum variation in neck tilt and these errors may be mimicked by rigid shifts that are simulated by the robust optimization. However, some anatomic changes may require a different robustness approach, such as deformation modeling within robust optimization or online adaptive treatment [17].

## Conclusion

Minimax optimized IMPT and IMRT plans were comparably robust in the presence of all simulated combinations of systematic range and setup errors, but PTV-based IMPT plans were not. The estimated clinical benefit in terms of adequate target coverage and minimal NTCP of minimax IMPT was in all HNC larger than IMRT, but in 7 especially substantial. The NTCP benefit of IMPT compared to IMRT remained similar in worst-case error scenarios. Increasing field numbers did not contribute to plan robustness, but contributed to organ sparing. Therefore, we conclude that minimax IMPT with a sufficient number of fields offers the opportunity to create robust plans with increased estimated clinical benefit compared to IMRT.

## Supporting Information

S1 Fig Target volumes and organ at risks objectives. (DOCX)

S1 Table Average optimization dose objectives used for IMRT and minimax IMPT. (DOCX)
