# Supplementary figures and images for: Robust Intensity Modulated Proton Therapy (IMPT) Increases Estimated Clinical Benefit in Head and Neck Cancer Patients
Source: PLoS One. 2016 Mar 31;11(3):e0152477. doi: 10.1371/journal.pone.0152477 (PMC4816406; doi:10.1371/journal.pone.0152477)

**S1 Figure. Target volumes and organ at risks objectives.**


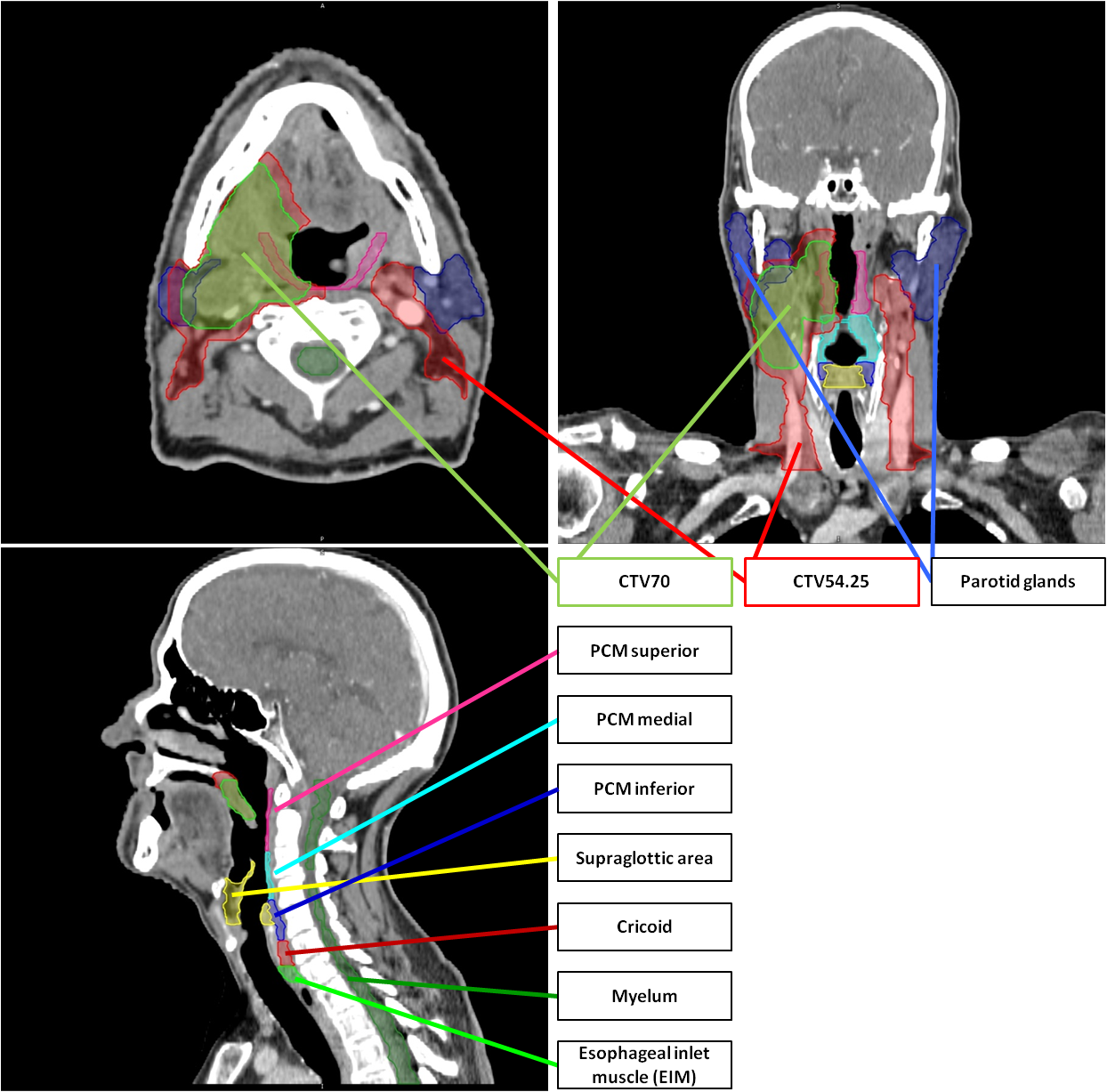

Supplement: S1 Fig — (DOCX) [file pone.0152477.s001.docx]
