# Supplementary material for: Robust Intensity Modulated Proton Therapy (IMPT) Increases Estimated Clinical Benefit in Head and Neck Cancer Patients
Source: PLoS One. 2016 Mar 31;11(3):e0152477. doi: 10.1371/journal.pone.0152477 (PMC4816406; doi:10.1371/journal.pone.0152477)
Supplement: S1 Table — (DOCX) [file pone.0152477.s002.docx]

**S1 Table. Average optimization dose objectives used for IMRT and minimax IMPT.**

|  | **IMRT** | | | | | | **Minimax IMPT** | | | | | | | |
| --- | --- | --- | --- | --- | --- | --- | --- | --- | --- | --- | --- | --- | --- | --- |
|  | objective | SD | weight | | SD | objective | | | SD | | weight | | SD | |
| **Parotid Li (Max EUD)** | 19.5 | ± 4.7 | 3.9 | ± 2.8 | | 14.4 | | ± 6.5 | | 5.8 | | ± 7.6 | |  |
| **Parotid Ri (Max EUD)** | 19.8 | ± 7.2 | 4.7 | ± 4.2 | | 19.7 | | ± 16.9 | | 7.2 | | ± 12.5 | |  |
| **PCM Sup (Max EUD)** | 43.8 | ± 15.3 | 1.8 | ± 3.1 | | 42.4 | | ± 11.2 | | 1.9 | | ± 3.1 | |  |
| **PCM Med (Max EUD)** | 44.1 | ± 11.7 | 1.4 | ± 0.9 | | 39.9 | | ± 10.3 | | 1.1 | | ± 0.8 | |  |
| **PCM Inf (Max EUD)** | 39.0 | ± 10.9 | 3.1 | ± 3.1 | | 31.6 | | ± 12.4 | | 3.3 | | ± 3.3 | |  |
| **Cricoid (Max EUD)** | 33.3 | ± 8.6 | 4.3 | ± 4.0 | | 25.8 | | ± 10.0 | | 4.0 | | ± 4.5 | |  |
| **EIM (Max EUD)** | 28.2 | ± 6.4 | 2.0 | ± 3.0 | | 22.0 | | ± 9.9 | | 3.0 | | ± 4.0 | |  |
| **Supraglottic (Max EUD)** | 39.9 | ± 8.9 | 0.9 | ± 0.4 | | 30.8 | | ± 11.6 | | 3.0 | | ± 3.7 | |  |

Objectives (in Gy) and weights (in arbitrary units) are given of all patients and the standard deviations. These parameters represent best values as selected by experienced treatment planning specialists in our department.

Abbreviation: PCM, pharyngeal constructor muscle; EIM, Esophageal inlet muscle; Max EUD, maximum equivalent uniform dose. Alpha was 1, thus EUD is equivalent to the mean dose.

Note: Dose objectives and weights were kept identical for all IMPT plans in all field configurations
